# 16S rRNA Amplicon Sequencing of Seawater Microbiota from Quintero Bay, Chile, Affected by Oil Spills, Shows the Presence of an Oil-Degrading Marine Bacterial Guild Structured by the Bacterial Genera *Alcanivorax, Cobetia, Halomonas*, and *Oleiphilus*

**DOI:** 10.1128/MRA.01366-18

**Published:** 2018-11-29

**Authors:** Claudia Ibacache-Quiroga, Juan Ojeda, M. Alejandro Dinamarca

**Affiliations:** a Centro de Micro-Bioinnovación (CMBi), Universidad de Valparaíso, Valparaíso, Chile; b Escuela de Nutrición y Dietética, Facultad de Farmacia, Universidad de Valparaíso, Valparaíso, Chile; University of Maryland School of Medicine

## Abstract

The Quintero Bay, located along the central coast of Chile, has suffered different oil spills during the past 5 years, impacting marine ecosystems. This report describes the microbial community structure of seawater samples obtained from the Quintero Bay through 16S rRNA amplicon sequencing.

## ANNOUNCEMENT

The Quintero Bay, located along the central Pacific coast of Chile, stands out due to its intense port and industrial activities, such as crude oil refining, thermoelectric generation, and mining. These activities occur close to urban areas and coexist with tourism, agriculture, aquaculture, and artisanal fishing. Since 2014, three major oil spills have occurred, generating a great social, ecological, and economic impact. This report describes the microbial community in seawater of two marine areas of the Quintero Bay through a 16S rRNA amplicon analysis.

Two seawater samples from the Quintero Bay were taken in autumn (23 May 2016) by a diver using sterile nuclease-free sampling bottles. Sample CGB1062-1 (10 m deep, 11°C) was taken near shipping docks (32°45′42″S, 71°29′44″W), while sample CGB1062-2 (10 m deep, 10°C) was sampled from the northwest area (32°44′05″S, 71°30′14″W). Samples were immediately preserved at 4°C for 1 h, and metagenomic DNA was extracted using a Meta-G-Nome DNA isolation kit (Epicentre). DNA integrity and concentration were analyzed by agarose electrophoresis and UV absorbance using the QuantiFluor fluorometer (Promega), respectively. The fourth hypervariable (V4) region of 16S rRNA was amplified from 10 ng of total DNA from each sample through quantitative PCR (qPCR), following the protocol developed by Caporaso and coworkers (1). The length, integrity, and distribution of the 16S metagenomic DNA libraries were estimated using a 2100 Bioanalyzer (Agilent Technologies), and they were sequenced using the Illumina MiSeq platform, with a read length of 2 × 150 bp, to generate paired-end reads that were filtered using Trimmomatic (v0.38) (2). Illumina adapter sequences and sequences with a Phred score of ≤30 were trimmed off, and quality-filtered sequences were merged. High-quality sequences were analyzed using Quantitative Insights into Microbial Ecology (QIIME, v1.9) (3). Operational taxonomic units (OTUs) were generated via open reference picking and identified and analyzed using the Greengenes database (v13.8). Nonassigned sequences were clustered against each other using *de novo* clustering. Clustering was performed using the UCLUST algorithm (4) with 97% identity. Each OTU was formed by at least five sequences, and one sequence per cluster was selected for further analysis.

Based on the amplicon analysis, the most abundant phylum in both samples was Proteobacteria (72.3% in CGB1062-1 and 56.2% in CGB1062-2), followed by Bacteroidetes (16.4% in CGB1062-1 and 16.2% in CGB1062-2). The most abundant orders in CGB1062-1 and CGB1062-2 were Rhodobacterales (28%) and Flavobacteriales (19.5%), respectively. Belonging to the phylum Proteobacteria, the most representative classes were Alphaproteobacteria and Gammaproteobacteria. From Gammaproteobacteria, Oceanospirillales was the most abundant order in both samples, in which the marine hydrocarbon-degrading genera Alcanivorax, Cobetia, Halomonas, and Oleiphilus were identified. Alcanivorax and Oleiphilus spp. are obligate aliphatic hydrocarbon-degrading marine bacteria (5, 6) considered active bioremediators after crude spills and bioindicators of polluted environments, while Cobetia spp. grow using aromatic or heterocyclic aromatic hydrocarbons (7).

This is the first report showing the seawater microbially diverse populations of the anthropogenically impacted Quintero Bay, with information for the exploration of new microbial resources and bioremediation of this area.

### Data availability.

The 16S rRNA metagenomic sequences of samples CGB1062-1 and CGB1062-2 were deposited in the NCBI Sequence Read Archive (SRA) under the accession numbers SRR7811726 and SRR7811770, respectively.
